# Establishing an Improved Cellular Photoaging Model by Repeated UVA Exposure of Human Skin Fibroblasts

**DOI:** 10.1111/srt.70309

**Published:** 2025-12-13

**Authors:** Wen‐Jun Tang, Zhuo‐Ran Li, Guang‐Lian Wang, Yu‐Fan Xue, Yi‐Yin Kou, Jia Zhao, Chi Zhang, Li‐Wen Li, Zhuan‐Li Bai

**Affiliations:** ^1^ Department of Plastic and Aesthetic Maxillofacial Surgery The First Affiliated Hospital of Xi'an Jiaotong University Xi'an China; ^2^ Zonglian College Xi'an Jiaotong University Xi'an China; ^3^ Key Laboratory of Resource Biology and Biotechnology in Western China Northwest University Ministry of Education Xi'an China

**Keywords:** fibroblast, ROS, senescence‐associated β‐galactosidase, skin photoaging, UVA

## Abstract

**Background:**

Repeated ultraviolet A (UVA) exposure‐induced cellular photoaging models had been widely applicated to study skin photoaging. However, there is no standard protocol to prepare these models and cell passaging is inevitably needed when fibroblasts are cultured in conventional media for 5–7 days.

**Materials and Methods:**

The adhesion ability of fibroblasts was tested after UVA irradiation. And both the cell culture medium and the UVA dose were optimized to help fibroblasts surviving a 7‐day culture period without cell subculture and to establishing an improved cellular photoaging model. Finally, senescence‐associated β‐galactosidase staining, intracellular reactive oxygen species (ROS) detection with the fluorescent redox probe dichlorodihydrofluorescein diacetate (DCFH‐DA), and Western blot analysis were performed to compare the improved model with the classical model.

**Results:**

UVA exposure induced a significant decrease in the adhesion ability of fibroblasts, and thus implied that cell passaging might be a screen pressure against the photodamaged cells. And fibroblasts incubated in the medium supplemented with 1% fetal calf serum could survive the 7‐day culture period without cell subculture and tolerate UVA irradiation up to a dose of 5.8 J/cm^2^ daily for 7 days. No significant differences were found between the improved model and the classical model in intracellular ROS production; however, our model demonstrated a significantly higher percentage of senescence‐associated β‐galactosidase positive cells. Moreover, both p53 and p21 were up‐regulated in our model, while in the classical model only p21 was up‐regulated.

**Conclusion:**

An improved cellular photoaging model was established, which seems to be more suitable than the classical model for elucidating the underlying molecular mechanisms of skin photoaging.

**Abbreviations**
DMEM: Dulbecco's Modified Eagle Medium
FCS: fetal calf serum
PBS: phosphate‐buffered saline
ROS: reactive oxygen species
SA‐*β*‐Gal: senescence‐associated *β*‐galactosidase
SDS‐PAGE: sodium dodecyl sulfate‐polyacrylamide gel electrophoresis
UVR: ultraviolet radiation

## 1 Introduction

Skin is the largest organ that defends the human body against environmental damages, such as sun exposure, air pollution and pathogen invasion [1]. Beyond natural chronological aging, the skin undergoes photoaging in consequence of ultraviolet radiation (UVR) from the sunlight [2, 3, 4]. Photoaging is a cumulative process and UVR exposure triggers a complex series of responses in human skin, ultimately resulting in clinical signs such as roughness, dryness, sagging, deep skin wrinkles, excessive pigmentation, and angiotelectasis [5, 6, 7]. Substantial reports have demonstrated that overproduction of reactive oxygen species (ROS), DNA damage, chronic inflammation, cellular senescence, and upregulation of matrix metalloproteinases (MMPs) are all associated with skin photoaging [7, 8, 9, 10, 11, 12, 13]. However, the underlying mechanisms of UVR‐induced photoaging remain to be fully elucidated.

To investigate the molecular mechanism of skin photoaging, both animal and cell models have been developed [1, 14, 15]. However, there had been no standard procedure to prepare these photoaging models, especially in vitro cellular photoaging models. Although substantial studies utilize a single ultraviolet A (UVA) exposure to establish the fibroblast photoaging model, repeated UVA exposure is considered to be more appropriate for recapitulating the cumulative nature of photoaging [16]. A variety of repeated UVA protocols have been reported, spanning different dosages and durations, such as three doses of 10 J/cm^2^ at Day 1, Day 4, and Day 6 weekly for 2 weeks [17], daily doses of 100 mJ/cm^2^ for 5 days [18], three doses of 5 J/cm^2^ with each exposure separated by 48‐h interval [16, 19], daily doses of 10 J/cm^2^ for 3 days [20], daily doses of 10 J/cm^2^ for 3 –14 days [21, 22, 23], two doses of 0.5–2 J/cm^2^ daily for 4 days [24], three doses of 6 J/cm^2^ daily for 4 days [25], and daily doses of 12 J/cm^2^ for 5 days [26]. Critically, these reports fail to address the necessity of cell subculture, which is typically required within 3–4 days when fibroblasts are maintained in classical complete medium.

The standard cell passage procedure utilizes trypsin to cleave the extracellular matrix (ECM) proteins essential for cell adhesion. Since fibroblasts undergoing UVA‐induced senescence demonstrate both compromised ECM synthesis and enhanced ECM degradation due to upregulation of MMPs [16, 27], thereby their adhesion and reattachment capabilities are severely impaired. We therefore hypothesize that the cell passage acted as a selection pressure, causing a differential loss of photodamaged cells and introducing bias into the established cellular models.

To eliminate the methodological bias that associated with cell subculture in the repeated UVA exposure models, we first investigated the effects of UVA radiation on the adherence capability of skin fibroblasts. Subsequently, we developed an improved cellular photoaging model that avoids cell subculture by optimizing the fibroblast culture conditions and the daily UVA exposure dose. Finally, we verified the effectiveness and characteristics of this improved model by comparing it with the classical model that previously reported.

## 2 Materials and Methods

### 2.1 Cell Culture

Human dermal primary fibroblasts were extracted from foreskin tissues procured through circumcision procedures of healthy male volunteers aged between 6 and 12 years after they had signed the written informed consent. Cells were cultured in Dulbecco's Modified Eagle Medium (DMEM, sourced from Gibco, Grand Island, NY, USA), with 10% fetal calf serum (FCS, Gibco) and 1% penicillin‐streptomycin solution (Genview, FL, USA) under conditions of 37°C and 5% CO_2_ in a humidified incubator. Fibroblasts ranging from the third to ninth passages were used to conduct further experiments. Experiments were repeated using cells derived from different subjects to ensure reproducibility. Primary fibroblasts were cultured into new culture plates at a seeding density of 3 × 10^3^ cells/cm^2^ once cellular confluence reached approximately 80%–90%, marked as one passage.

### 2.2 UVA Irradiation

UV TL‐K lamps (Philips, Eindhoven, Netherlands), with an emission wavelength range spanning from 320 to 400 nm (peak at 365 nm), served as the source of UVA irradiation, and the dosage of UVA was precisely quantified by UVA radiometer (Sigma, Shanghai, China). Before UVA irradiation, medium was removed from cell cultures. Then cells were washed twice with phosphate‐buffered saline (PBS) and covered with a thin layer of PBS. Fibroblasts were exposed to a defined dose of UVA irradiation once daily for seven consecutive days. After UVA irradiation, the PBS was immediately changed to fresh medium, and cells were cultured under conditions of 37°C and 5% CO_2_ in a humidified incubator. After 7 days of UVA irradiation, cells were collected for further experiments.

### 2.3 Assessment of Fibroblast Adhesion Ability

Fibroblasts of the photoaged group were exposed to UVA irradiation at a total dose of 10 J/cm^2^; fibroblasts of H_2_O_2_ group were treated with 3% H_2_O_2_ for 4 h and fibroblasts of the normal group with no intervention served as control. Then fibroblasts were subcultured at a ratio of 1:2. Cells were imaged after 1, 2, and 4 h, respectively. The number of adherent cells was counted in a 200 µm × 200 µm square. The experiments were performed in triplicate and data were represented as means ± SD.

### 2.4 Cell Viability Assay

The Cell Counting Kit‐8 (CCK‐8; NCM, Suzhou, China) assay was used to determine cell viability. Cells were seeded at a density of 5 × 10^3^ cells per well in 96‐well culture plates and incubated overnight within the humidified chamber. Then the culture media were changed to DMEM supplemented with different concentrations of FCS (0.25%, 0.5%, 1%, 2%, 5% and 10%) for 7 days. Following this treatment period, cell number was counted. Then 10 µL of CCK‐8 reagent was added to each well, and the plates were incubated at 37°C for 2 h. The absorbance was then quantified at 450 nm using fluorescence microplate reader. The sample of fibroblasts cultured with 10% FCS supplemented DMEM that freshly reached full confluence was used as a control and the formula to calculate cell viability is (Absorbance of cells cultured for 7 days/Absorbance of fibroblasts freshly reached full confluence) × 100%. All experiments were performed in triplicate.

### 2.5 Senescence‐Associated β‐Galactosidase Staining

The activity of senescence‐associated β‐galactosidase (SA‐*β*‐Gal) was quantified following the protocols supplied with the β‐galactosidase staining kit (C0602, Beyotime, Shanghai, China). Cells were rinsed in PBS and then fixed for 15 min at room temperature (RT) with a fixative solution. Afterward, cells were incubated with the staining solution at 37°C overnight. The percentage of SA‐*β*‐Gal‐positive cells was determined by counting the number of positively stained cells relative to the total cell count across four continuous visual fields under a microscope (total magnification: 200×).

### 2.6 Flow Cytometry

Cells were cultured in DMEM supplemented with either 1% or 0.5% serum for 7 days before experiment. Subsequently, the cells were collected, centrifuged at 252 × *g* for 10 min at RT, and washed three times with cold PBS. FITC Annexin V apoptosis detection kit (V13241, Invitrogen; Thermo Fisher Scientific, Inc.) was used for apoptosis detection. Cells were resuspended in 100 µL of Annexin V binding buffer. Five microliters of FITC Annexin V was added, and incubated for 15 min at RT in dark. Subsequently, 200 µL of binding buffer with propidium iodide concentration of 1 µg/mL was added to each tube and incubate for 10 min in the dark, then the fluorescent signatures of at least 1 × 10^4^ cells were immediately evaluated using FL2 channel with a flow cytometer (BD Biosciences).

Following 7 days of incubation at 37°C with 5% CO_2_, the cells were stabilized with 70% cold ethanol and then stained with 50 µg/mL propidium iodide (Thermo Fisher Scientific, Inc.) and 100 µg/mL RNase A (Thermo Fisher Scientific, Inc.) for 30 min in the dark. The cell cycle was assessed using FL3 channel with a flow cytometer (BD Biosciences), and analyzed using FlowJo software v10. And cells cultured under the routine condition was used as control.

### 2.7 Assessment of Intracellular ROS Production

Intracellular ROS were determined with 2′,7′‐dichlorodihydro‐fluoresce in diacetate (DCFH‐DA; Beyotime, Shanghai, China). Prior to the assay, cells in 6‐well plates were exposed to varying doses of UVA irradiation daily for 7 days. Then cells were rinsed with PBS and incubated in 1 mL of 10 µM DCFH‐DA solution for 30 min in the dark. Cells were then collected into 1.5 mL centrifuge tubes, washed twice with PBS and centrifuged at 252 × g for 5 min, aspirated the supernatant thoroughly, and then resuspended the cells in PBS. The fluorescence intensity of at least 1 × 10^4^ cells per sample was measured using the fluorescence microplate reader, setting the optimal excitation wavelength at 488 nm and the optimal emission wavelength at 525 nm. All experiments were performed in triplicate.

### 2.8 Western Blotting

The total protein of cells was obtained using RIPA buffer mixed with 1% phenylmethylsulfonyl fluoride (PMSF), and the protein concentration was quantified with a BCA kit (NCM, Suzhou, China). Twenty micrograms protein of each sample was loaded onto 10% sodium dodecyl sulfate‐polyacrylamide gel electrophoresis (SDS‐PAGE) gels, separated by electrophoresis, and then transferred to polyvinylidene fluoride (PVDF) membranes. The PVDF membranes were blocked in a solution of 5% non‐fat milk in TBST for 60 min at room temperature and then incubated with the secondary antibody diluted at 1:2000 in 5% non‐fat milk in TBST for 120 min at room temperature after binding to the specific primary antibody. The primary antibodies included p53 (7F5, Cell Signaling; MA, USA), p21 Waf1/Cip1 (12D1, Cell Signaling; MA, USA), GAPDH (14C10, Cell Signaling; MA, USA). All the primary antibodies were diluted at 1:1000, and incubated with membranes overnight at 4°C. The signals were detected using a chemiluminescence detection system (Sinsage. Beijing, China) and analyzed using ImageJ software (NIH, Bethesda, MD, USA).

### 2.9 Statistical Analysis

Data are expressed as mean ± SD. Statistical analyses were performed using Prism software v.5.0.3 (GraphPad Prism, San Diego, CA, USA). The determination of statistical significance was achieved through the application of normal distributions, one‐way analysis of variance (ANOVA) tests. All experiments were conducted in triplicate, and the statistical means and standard deviations were calculated, significance was established at *p* value less than 0.05.

## 3 Results

### 3.1 UVA Irradiation Attenuates Cell Adhesion Ability

**FIGURE 1:**
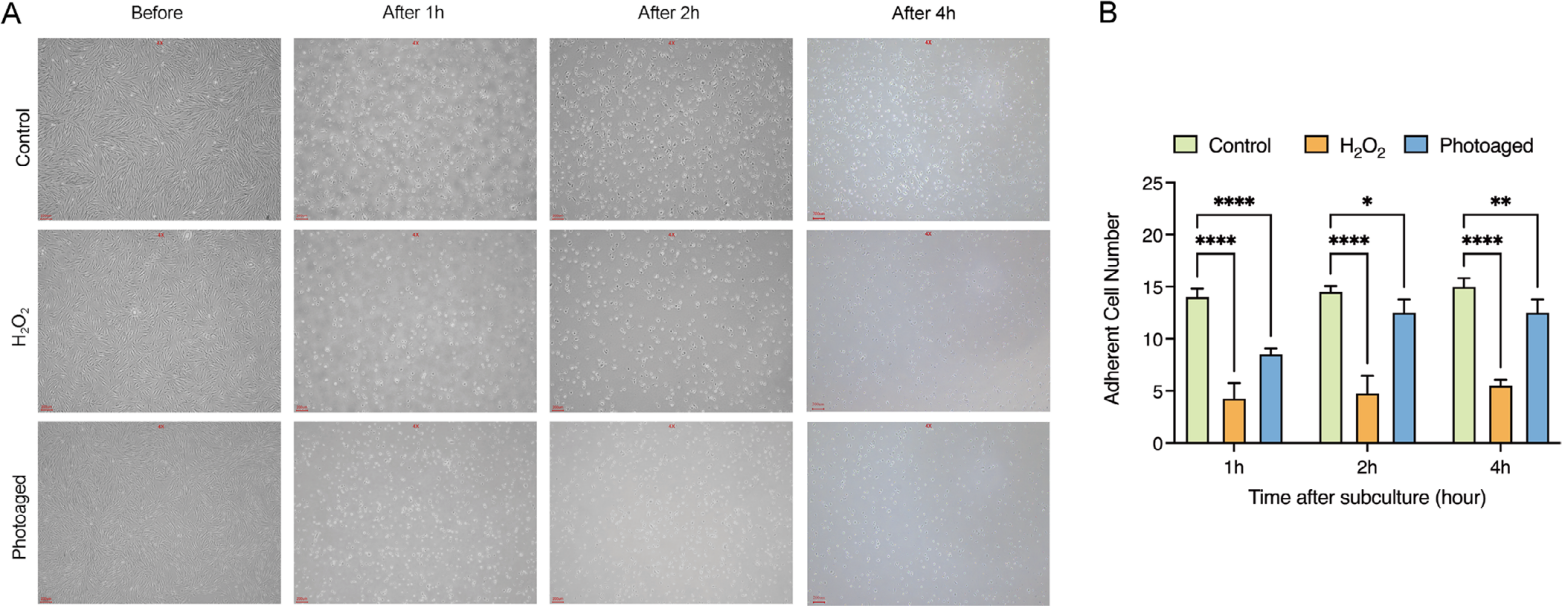
∣ Assessment of fibroblast adhesion ability after cell subculture. (A) Representative photographs of the attached UVA‐irradiated cells and H_2_O_2_‐treated cells after cell subculture at a ratio of 1:1. (B) Quantification of the number of adherent cells per 0.04 mm^2^ (200 µm × 200 µm) after cell subculture. Control: normal cells without intervention; Photoaged, UVA‐irradiated cells; H_2_O_2_: H_2_O_2_‐treated cells. *, *p* < 0.05; **, *p* < 0.01; ****, *p* < 0.0001.

**FIGURE 2:**
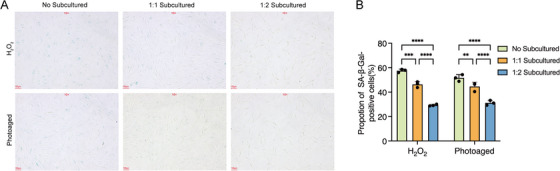
∣ SA‐*β*‐Gal staining after cell subculture. (A) Representative photographs of SA‐*β*‐Gal staining in UVA‐irradiated and H_2_O_2_‐treated cells after cell subculture at a ratio of 1:1 or 1:2. (B) The percentage of SA‐*β*‐Gal positive cells following cell subculture. Photoaged, UVA‐irradiated cells; H_2_O_2_: H_2_O_2_‐treated cells. **, *p* < 0.01; ***, *p* < 0.001; ****, *p* < 0.0001.

Previous report has demonstrated that cellular senescence is closely associated with a hyper‐adhesive cell phenotype with low motility [28]; however, whether passaged senescent cells could re‐attach to the bottom of the culture disk just as the normal cells do remains unknown. Cellular senescence was induced by H_2_O_2_ treatment or by exposure to UVA (10 J/cm^2^) and after cell subculture at a ratio of 1:2 for 1, 2, and 4 h, then the number of adherent fibroblasts per 0.04 mm^2^ (200 µm × 200 µm) was counted to assess cell adhesion ability. It was found that the number of the attached cells of the control group was significantly higher than that of H_2_O_2_‐treated group and UVA‐irradiated group at all the three time points, indicating that the adhesion ability of senescent fibroblasts was remarkably impaired when compared with normal fibroblasts (Figure 1A). Moreover, the number of the adherent cells from UVA‐irradiated group was significantly increased from 1 to 2 h after cell passaging (8.50 ± 0.58 vs. 12.50 ± 1.29, *p < *0.001), while no significance was found between the adherent cell number from 2 and 4 h (12.50 ± 1.29 vs. 12.50 ± 1.29, *p > *0.05) (Figure 1B). Furthermore, cellular senescence was determined by SA‐*β*‐Gal staining (Figure 2A). And we found that the percentage of SA‐*β*‐Gal positive cells of the H_2_O_2_‐treated group decreased from 57.49 ± 1.36% to 46.34 ± 2.34% (*p < *0.001) and 29.31 ± 0.72% (*p < *0.0001) after 1:1 or 1:2 cell subculture (Figure 2B), respectively, and the percentage of SA‐*β*‐Gal positive cells of the UVA‐irradiated group decreased from 51.60 ± 2.73% to 44.60 ± 3.61% (*p *< 0.01) and 31.17 ± 1.98% (*p < *0.0001) after 1:1 or 1:2 cell subculture (Figure 2B), respectively, implying that cell passage led to a significant loss of the aging cells.

These results indicated that UVA exposure could attenuate the adhesion ability of fibroblasts and cell passaging might be a selecting pressure against photodamaged cells. Since skin photoaging is a chronic and cumulative process, cell subculture should be avoided in the procedure of establishing cellular models to recapitulate the aspects of the disease.

### 3.2 Optimizing FCS Concentration

**FIGURE 3:**
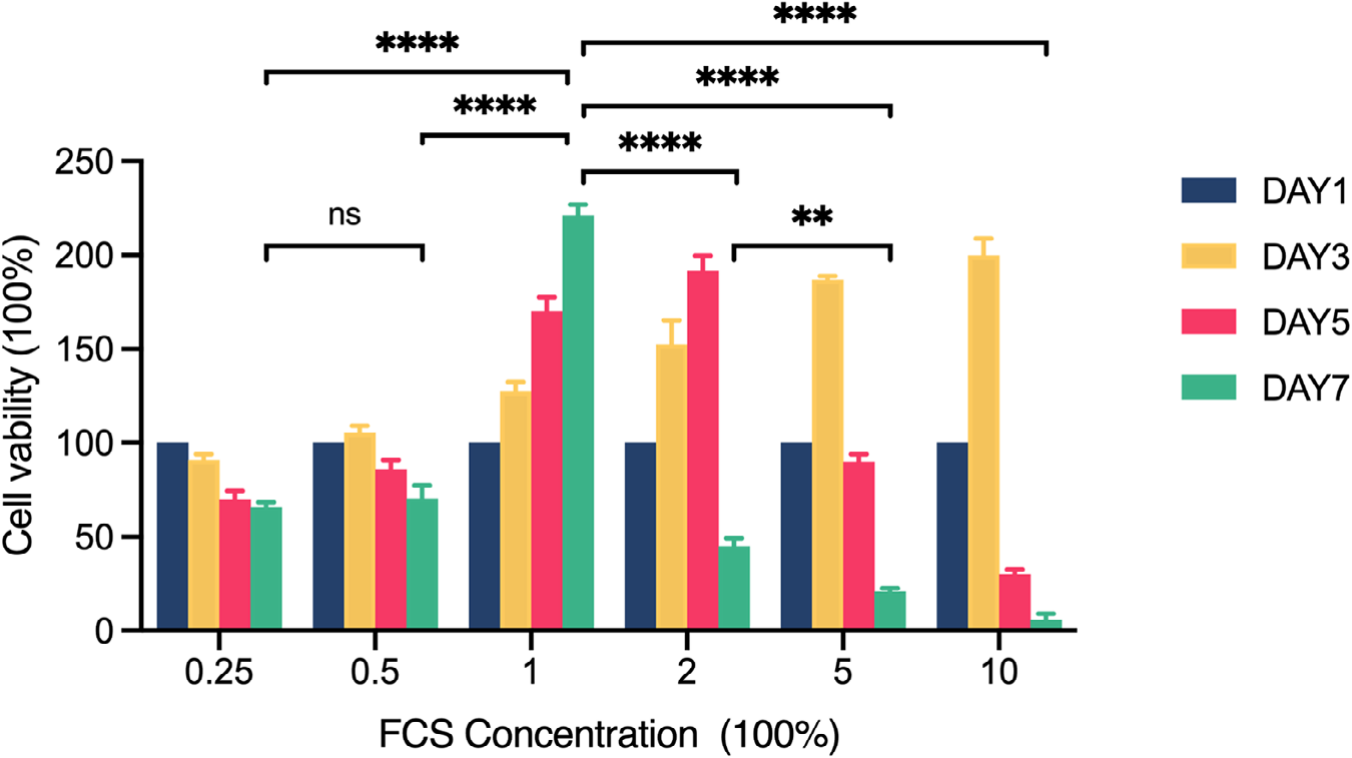
∣ CCK‐8 cell viability assessment. CCK‐8 assay results showing cell viability for fibroblasts cultured with different concentrations of FCS. **, *p* < 0.01; ***, *p* < 0.001; ****, *p* < 0.0001; ns, *p* > 0.05.

**FIGURE 4:**
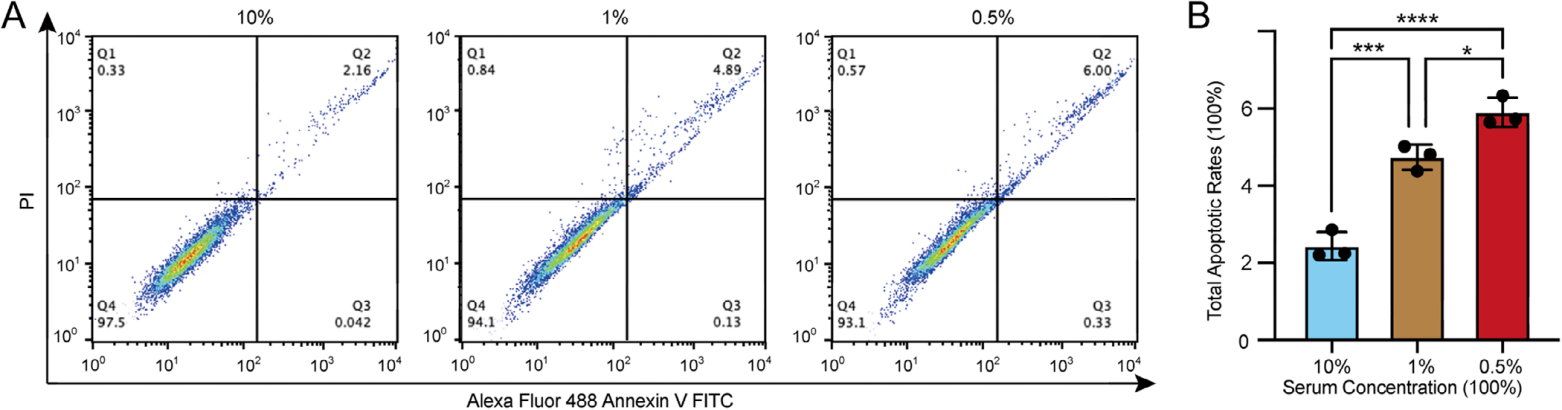
∣ Cellular apoptosis assay of the fibroblasts cultured with different concentration of FCS. (A) Flow cytometry analysis using Annexin V‐PI staining to evaluate the percentage of apoptotic cells. (B) Quantification of the total apoptotic rates of the fibroblasts cultured with different concentration of FCS. *, *p* < 0.05; ***, *p* < 0.001; ****, *p* < 0.0001.

**FIGURE 5:**
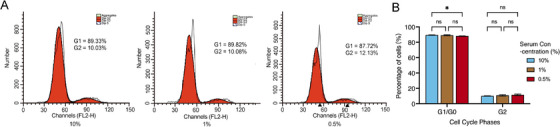
∣ Cell cycle analysis of the fibroblasts cultured with different concentration of FCS. (A) Cell cycle analysis performed by flow cytometry following propidium iodide staining. (B) Quantification of the percentage of cells in different cell cycle phases. *, *p* < 0.05; ns, *p* > 0.05.

To prevent the loss of photodamaged cells during cell passaging, the concentration of FCS supplemented to fibroblast culture medium was optimized since FCS concentration plays a key role in maintaining cell growth. Cells were seeded at a density of 5 × 10^3^ cells per well in 96‐well culture plates and cultures in the media supplemented with different concentration of FCS. After 7‐day culture without changing medium or subculture, cell viability decreased significantly in along with the increase of FCS concentration when it was higher than 1% (Figure 3), which might due to the death of fibroblasts resulting from cell over‐proliferation induced nutritional insufficiency. And cell viability was lower when cells were cultured with 0.25% or 0.5% FCS in comparison with those cultured with 1% FCS (Figure 3), while no statistical significance was found between 0.25% and 0.5% FCS, suggesting that FCS concentration lower than 1.0% could not be enough to support fibroblast growth. Moreover, when compared with the cells cultured in 10% FCS supplemented medium, the ratio of cell apoptosis increased significantly (Figure 4A,B), while the ratio of cell populations in the G0/G1 or G2 phase remained unchanged in fibroblasts cultured with lower FCS concentrations (Figure 5A,B). Since the apoptosis rate of the cells cultured in 0.5% FCS supplemented medium was higher than that of the cells cultured in 1% FCS supplemented medium (Figure 4,B), fibroblasts were cultured in 1% FCS supplemented medium in the following experiment to omit subculture during a 7‐day modeling period. And cell counting assay demonstrated that fibroblasts seeded at an initial density of 5 × 10^4^/cm^2^, reached a final density of about 1.2 × 10^5^/cm^2^ after 7‐day culture with the optimized condition.

### 3.3 Optimizing the Dose of UVA Irradiation

**FIGURE 6:**
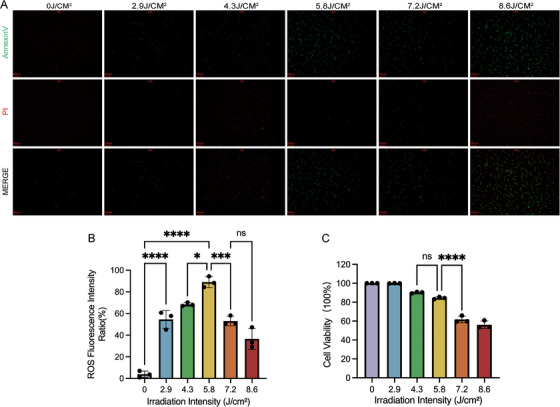
∣ Optimization of UVA irradiation dosage. (A) Cell death analysis determined by FITC‐Annexin V‐PI staining. (B) The fluorescence intensity of ROS production assessed by DCTH‐DA probe‐based flow cytometry assay. (C) Cell viability assessment using the CCK‐8 assay. *, *p* < 0.05; ***, *p* < 0.001; ****, *p* < 0.0001; ns, *p* > 0.05.

It is generally accepted that UVA‑mediated ROS generation leads to oxidative stress and ultimately results in skin photoaging. Primary fibroblasts cultured with 1% FCS supplemented DMEM were exposed to different dose of UVA irradiation daily for 7 days. Then intracellular ROS generation was determined using a DCFH‐DA probe‐based fluorescence assay (Figure 6A). And we found that the percentage of fluorescent cells elevated with the increase of UVA dosage (0 J/cm^2^, 3.89% ± 2.99%; 2.9 J/cm^2^, 54.5% ± 8.3%; 4.3 J/cm^2^, 68.39% ± 1.66%; 5.8 J/cm^2^, 88.96% ± 5.07%); however, the percentage of fluorescent cells decreased markedly when the UVA dose is over 5.8 J/cm^2^ (Figure 6B), implying massive cell death occurred since dead cells do not have ROS‐generating metabolic processes. To confirm massive cell death was induced when the UVA dose was over 5.8 J/cm^2^, CCK8 cell viability assay was performed and a sudden decrease in cell viability was observed when UVA dose reached 7.2 J/cm^2^ (Figure 6C). And FITC‐Annexin V–PI staining showed a significant increase in the number of both early apoptotic cells (green stained) and dead cells (red stained) when UVA dose reached 7.2 J/cm^2^ (Figure 6A) These results indicated that fibroblasts cultured with 1% FCS supplemented DMEM could tolerate UVA irradiation up to a dose of 5.8 J/cm^2^ daily for 7 days, which is obviously lower than most of the daily dose that used in establishing the classical UVA‐induced cellular photoaging model.

### 3.4 UVA Irradiation Induces Fibroblast Aging

**FIGURE 7:**
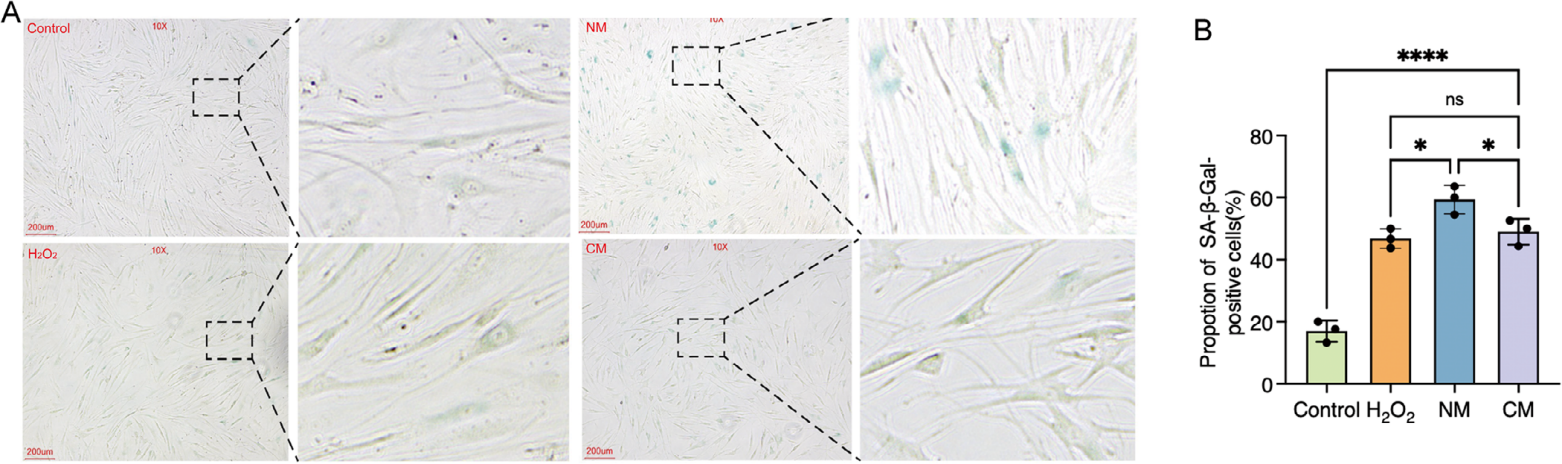
∣ Detection of senescent cells. (A) SA‐*β*‐Gal staining of fibroblasts after different treatments. (B) Quantification of the percentage of SA‐*β*‐Gal positive senescent cells. Control, normal cells; H_2_O_2_, H_2_O_2_‐treated group; NM, new model group; CM, classical model group. *, *p* < 0.05; ****, *p* < 0.0001; ns, *p* > 0.05.

Primary fibroblasts were divided into four groups, including the new model (NM) group, in which the cells were cultured with 1% FCS supplemented DMEM and subjected to 5.8 J/cm^2^ UVA irradiation daily for 7 days without cell subculture; the classical model (CM) group, in which the cells were cultured with 10% FCS supplemented DMEM and subjected to 10.0 J/cm^2^ UVA irradiation daily for 7 days with cell subculture at Day 4; the positive control group, in which the cells were cultured with 10% FCS supplemented DMEM and treated with 3% H_2_O_2_ for 4 h to induce cellular senescence; and the normal control group, in which the cells were cultured with 10% FCS supplemented DMEM for 24 h post cell passaging. Since the activity of SA‐*β*‐Gal is one of the most widely used the biomarker of aging, all the samples were subjected to the SA‐*β*‐Gal staining to assess cellular senescence. Although SA‐*β*‐Gal positive cells were observed in both the UVA‐irradiated groups (Figure 7A), the proportion of the positive cells was significantly higher in NM group than that of the CM group (59.37 ± 4.58% vs. 49.00 ± 4.19%, *p *< 0.05, Figure 7B), supporting that senescent cells were lost during cell passaging.

### 3.5 UVA Eradiation Promotes Intracellular ROS Generation

**FIGURE 8:**
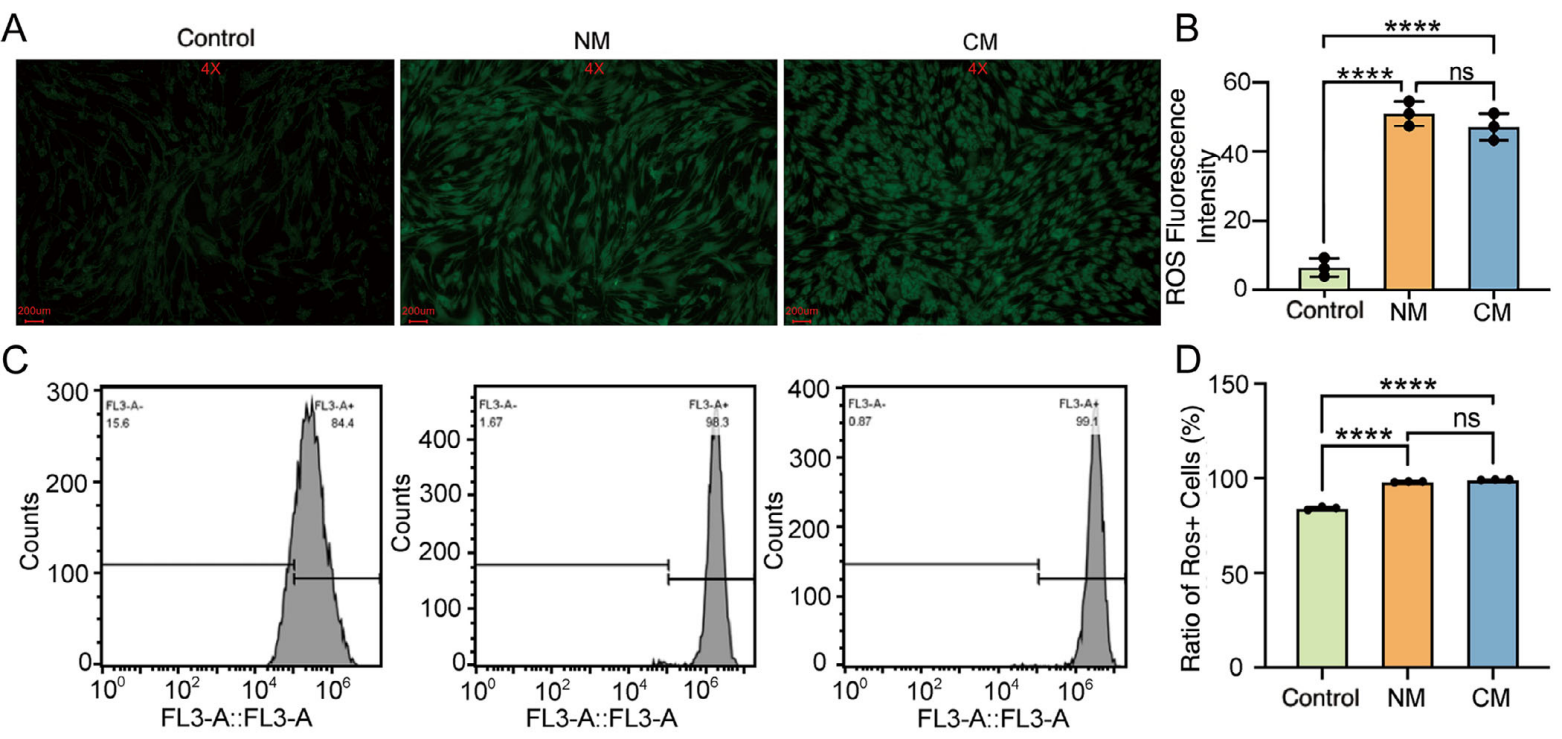
∣ Assessment of intracellular ROS production. (A) Representative photographs of DCTH‐DA probe‐stained cells (scale bars 200 µm). (B) Quantitative analysis of relative fluorescence intensities. (C) Flow cytometry assay showing ROS fluorescence intensity histograms. (D) Percentage of fluorescent cells. Control, normal cells; NM, new model group; CM, classical model group. ****, *p* < 0.0001; ns, *p* > 0.05.

To further elucidate the difference between NM and CM, intracellular ROS generation was assessed by a DCFH‐DA probe‐based fluorescence assay. As shown in Figure 8A, fibroblasts cultured under routine conditions showed light green fluorescence, but the UVA‐irradiated fibroblasts of both the NM group and the CM group showed bright fluorescence. Moreover, both the relative fluorescence intensity and the percentage of fluorescence positive cells in the NM and CM group were higher than that in the normal control group ((Figure 8B–D), while no statistical significance were found between the NM group and the CM group. These results indicated that UVA exposure promoted intracellular ROS production.

### 3.6 Repeated UVA Exposure Activates p53‐p21 Axis

**FIGURE 9:**
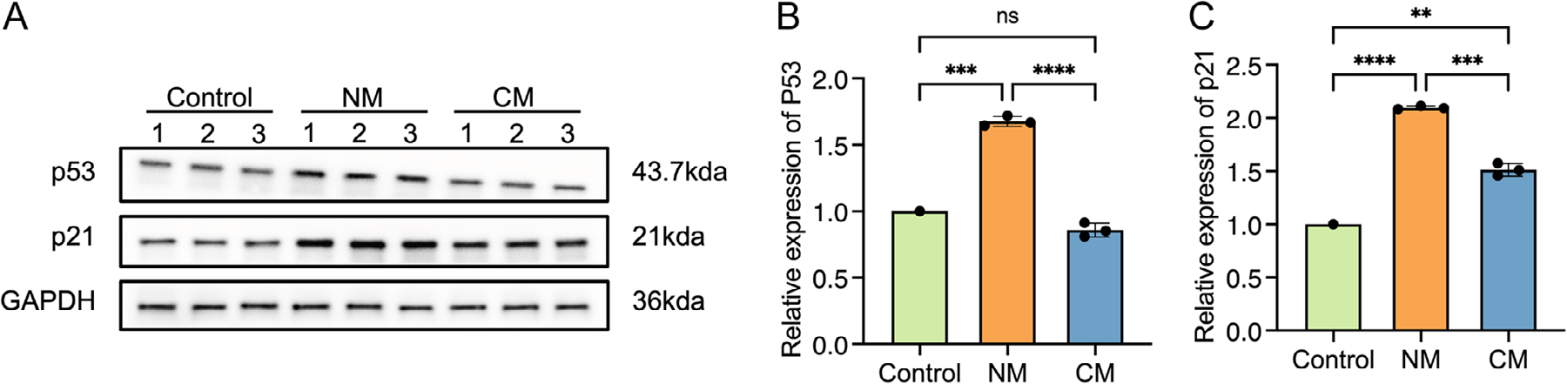
∣ Western blot analysis of p53 and p21. (A) Western blots of total cellular extracts probed with antibodies against p53, p21, and GAPDH. (B) Analysis of relative p53 expression levels. (C) Analysis of relative p21 expression levels. Control, normal cells; NM, new model group; CM, classical model group. **, *p* < 0.01; ***, *p* < 0.001; ****, *p* < 0.0001; ns, *p* > 0.05.

Activation of p53‐p21 axis has been considered as one of the general hallmarks of cellular senescence [29]. Western blot analysis was performed to analyze the protein expression levels of p53 and p21 (Figure 9A). In comparison to that of the control group, the levels of both p53 and p21 were upregulated in the NM group, and only the level of p21 was upregulated in the CM group (Figure 9B,C). Moreover, the p21 level of the NM group was significantly higher than that of the CM group (Figure 9C). Surprisingly, these results implied that in NM group activation of p53‐p21 pathway is involved in UVA‐induced cellular senescence, while in CM group, cellular photoaging might be activated by a p53‐independent upregulation of p21.

## 4 Discussion

Skin photoaging, defined as the premature aging of the skin, is generally considered caused by repeated exposure to UVR from sun or artificial sources, since numerous reports demonstrate that UVR exposure induces pathological dermal alterations and sunscreens effectively prevent the process [8, 30]. Consequently, several repeated UVA exposure‐induced cellular models have been developed to investigate the molecular mechanisms of skin photoaging and to screen active anti‐photoaging ingredient from traditional Chinese medicinal herbs [16, 18, 19, 20, 21, 24, 25, 26, 31]. In these models, dermal fibroblasts are typically exposed to a defined daily dose of UVA irradiation for 3–14 days to recapitulate the disease. However, when fibroblasts are maintained in a conventional complete medium, cell passage is inevitably required during the experimental period. And no previous studies have investigated whether this procedure exerts an adverse bias on the experimental results.

In this study, we first evaluated the effects of UVR on cell adherence. Our results demonstrated that even a single exposure to UVA radiation significantly reduced the adhesion ability of skin fibroblasts. Moreover, a remarkable loss of SA‐*β*‐Gal positives cells was observed after cell subculture. Previous reports demonstrated that UV radiation induces transcriptome‐wide RNA damage through photochemical reactions generating pyrimidine dimers which result in ribosomes stall and global protein synthesis suppression [32, 33]. In addition, ribosome stalling can activate the p38 and JNK mitogen‐activated protein kinases through activation of ribotoxic stress response and ultimately led to the up‐regulation of MMPs [26, 34, 35, 36, 37]. Thereby it appears that both impaired ECM synthesis and enhanced ECM degradation contribute to the UVA‐induced loss of senescent cells during cell subculture. To overcome this methodological disadvantage, we established a new cellular photoaging model by maintaining primary fibroblasts in 1% FCS‐supplemented medium and irradiating them with a daily UVA dose of 5.8 J/cm^2^ for seven consecutive days, entirely eliminating the need for cell subculture.

The new cellular photoaging model developed in this work exhibits several significant differences compared to the previously reported classical model. Firstly, the new model showed a higher percentage of SA‐*β*‐Gal positive cells and a higher apoptotic cell rate than the classical model. This disparity suggests that photodamaged cells induced by UVA radiation, whether undergoing apoptosis or senescence, had an impaired ability to adhere and were screened out during cell subculture required to establish the classical model. Secondly, fibroblasts from the new model tolerated a lower dosage of UVA irradiation compared to cells from the classical model. This reduced tolerance is likely a result of the lower FCS concentration supplemented to the culture medium of the new model. Thirdly, the new model demonstrated upregulation of both p53 and p21, whereas the classical model exhibited upregulation of p21. Although early activation of p53‐p21 pathway is observed in both DNA damage response‐induced senescence and ROS‐induced senescence [29, 38], the regulatory effect of UVA on p53 expression in skin fibroblasts is controversial. Some reports indicate that p53 expression levels increase after a single or repeated exposure to UVA radiation [20, 21, 22, 39], yet others show that p53 phosphorylation is upregulated while the expression levels remained unchanged [24, 40]. This controversy may largely be attributed to the difference in time points used for harvesting UVA‐irradiated cells, given that p53 is a short‐lived protein with a half‐life of approximately 5–20 min [41]. However, in this study, we attribute the difference in p53 expression levels between the new model and classical model, at least partly, to the significant loss of photodamaged cells during the cell passage process.

Regarding the p53‐independent p21 upregulation observed in the classical model, previous reports have established that UVA irradiation promotes the production of latent TGF‐β1 and the expression of IL‐1 [42, 43, 44, 45]. Both TGF‐β1 and IL‐1 are known to upregulate p21 in a p53‐independent manner [46, 47]. Nevertheless, further investigation is required to determine whether TGF‐β1 and IL‐1 play critical roles in mediating p53‐independent p21 upregulation in UVA‐irradiated dermal fibroblasts.

One limitation of this study is the exclusive use of foreskin‐derived fibroblasts. Although these cells are widely employed in establishing cellular photoaging models, further investigation utilizing cells derived from different anatomical sites and female donors is required to fully validate the model's generalizability.

In our opinion, the new model seems more suitable than the classical model in recapitulating in vivo skin photoaging for no photodamaged cells were screened out during the process of model establishment. However, the FCS concentration and the dose of UVA radiation must be re‐optimized when using cells from other origins or different cell types. In addition, further optimization should be carried out to minimize the cell proliferation rate to mimicking the truly in vivo skin microenvironment.

## 5 Conclusion

A new cellular model of skin photoaging is established in this study by optimizing both the concentration of FCS supplemented to fibroblast culture medium and the dosage of UVA irradiation, which seems to have some advantages over the classical model in recapitulating skin photoaging by preventing cell subculture‐induced loss of photodamaged cells. And the new model might be widely applied to elucidating the underlying mechanisms of skin photoaging, screening anti‐photoaging medicines and developing novel strategies to prevent or alleviate cutaneous photoaging.

## Ethics Statement

The entire study was carried out in strict accordance with protocols approved by the Ethics Committee of The First Affiliated Hospital of Xi'an JiaoTong University (Approval number: LLSBPJ‐2024‐242).

## Conflicts of Interest

The authors declared no potential conflicts of interest with respect to the research, authorship, and/or publication of this article.

## Data Availability

The data that support the findings of this study are available from the corresponding author upon reasonable request.
